# Case report: A novel homozygous histidine triad nucleotide-binding protein 1 mutation featuring distal hereditary motor-predominant neuropathy with rimmed vacuoles

**DOI:** 10.3389/fneur.2023.1007051

**Published:** 2023-02-06

**Authors:** Nan Jiang, Rocio Vazquez Do Campo, Mohamed Kazamel

**Affiliations:** Division of Neuromuscular Disease, Department of Neurology, The University of Alabama at Birmingham, Birmingham, AL, United States

**Keywords:** charcot-marie-tooth (CMT), HINT1 mutation, neuromyotonia, rimmed vacuoles, motor-predominant neuropathy

## Abstract

**Introduction:**

Recessive mutations in the gene encoding the histidine triad nucleotide-binding protein 1 (HINT1) are associated with axonal motor-predominant Charcot–Marie–Tooth (CMT) disease with neuromyotonia. A total of 24 *HINT1* gene mutations have been reported so far. Some of these cases had mild to moderate elevations of creatinine kinase with no earlier reports of muscle biopsy findings in these cases. In this study, we describe a patient with axonal motor-predominant neuropathy and myopathy with rimmed vacuoles, likely due to a novel *HINT1* gene mutation.

**Case report:**

A 35-year-old African American man presented with insidious onset and progressive symmetric distal leg weakness followed by hand muscle atrophy and weakness since the age of 25. He had no muscle cramps or sensory complaints. His 38-year-old brother developed similar symptoms beginning in his early 30 s. On neurologic examination, the patient had distal weakness and atrophy in all limbs, claw hands, pes cavus, absent Achilles reflexes, and normal sensory examination. Electrodiagnostic studies revealed absent/reduced compound motor action potential amplitudes distally with normal sensory responses with no neuromyotonia. His sural nerve biopsy showed a chronic non-specific axonal neuropathy, and a biopsy of the tibialis anterior muscle demonstrated myopathic features and several muscle fibers harboring rimmed vacuoles without inflammation in addition to chronic denervation changes. A homozygous variant, p.I63N (c.188T > A), in the *HINT1* gene was found in both brothers.

**Conclusion:**

We describe a novel, likely pathogenic, *HINT1* pI63N (c.188T > A) homozygous variant associated with hereditary axonal motor-predominant neuropathy without neuromyotonia in two African American brothers. The presence of rimmed vacuoles on muscle biopsy raises the possibility that mutations in the *HINT1* gene may also cause myopathy.

## Introduction

Hereditary neuropathies are progressive disabling diseases with a wide variety of phenotypes and genotypes, with an estimated prevalence of one in 2,500 individuals worldwide (1) and 10–28 in 1,00,000 individuals in Europe (2). Charcot–Marie–Tooth (CMT) disease, also known as hereditary motor and sensory neuropathy, was first described by French neurologists Charcot and Marie (3) and a British neurologist Tooth in 1886 (4); it is the most common inherited neuropathy. The clinical picture encompasses distal muscle weakness and atrophy, foot deformities such as pes cavus, hand deformities such as claw hands, sensory abnormalities, and reduced or absent reflexes. Distal hereditary motor neuropathy (dHMN) is a less common variant of CMT characterized by motor-predominant axonal involvement with absent or minimal sensory deficits (5, 6). Mutations in more than 100 genes have been associated with CMT (7). CMT disease can be sub-classified according to median motor conduction velocity into demyelinating (< 35 m/s), intermediate (35–45 m/s), and axonal (>45 m/s) forms. Zimon et al. (8) reported that recessive loss-of-function mutations in the gene encoding the histidine triad nucleotide-binding protein 1 (*HINT1*) are associated with a specific form of CMT with neuromyotonia also known as autosomal recessive axonal neuropathy with neuromyotonia. Despite the increasing number of patients with *HINT1* being diagnosed worldwide, it remains challenging to assess the pathogenicity of novel variants. Here, we report a novel, likely pathogenic, *HINT1* pI63N (c.188T>A) homozygous variant associated with dHMN/axonal motor-predominant neuropathy in two African American brothers. We also report histopathologic myopathic features including rimmed vacuoles in the muscle biopsy of the proband, raising the possibility that mutations in *HINT1* gene may cause myopathy in addition to the neuropathy.

## Case description

A 35-year-old African American man with no significant past medical history was referred to our neuromuscular clinic for frequent falls and concern for neuropathy. He first noticed a bilateral foot drop causing an abnormal gait and frequent falls at the age of 25. He experienced slowly progressive muscle weakness with the involvement of the hands by the age of 33. He had no sensory complaints and no muscle cramping or stiffness. His 38-year-old brother also had a bilateral foot drop that started in his 30s and bilateral hand weakness. There was no history of a similar condition in any other family members, including parents and two paternal half-sisters. His neurologic examination revealed normal cognition and cranial nerve function without tongue weakness, atrophy, or fasciculation. Manual muscle testing revealed normal muscle strength in Medical Research Council grades except for finger extension 4/5, distal finger flexion 4/5, thumb abduction 3/5, hand interosseous muscles 4/5, hip flexion 5/5, ankle dorsiflexion 3/5, and ankle plantar flexion 4/5 bilaterally. He had muscle atrophy distally in all limbs with claw hands and pes cavus. Deep tendon reflexes were 2+ throughout except for absent Achilles tendon reflexes bilaterally. There was no percussion or handgrip myotonia, and the muscle tone was normal. Sensory examination was normal to pinprick, vibratory, and proprioceptive testing. He had a slow high steppage gait. He had bilateral contracture in the Achilles tendons with no gross spine deformity.

## Diagnostic assessment

**Figure 1:**
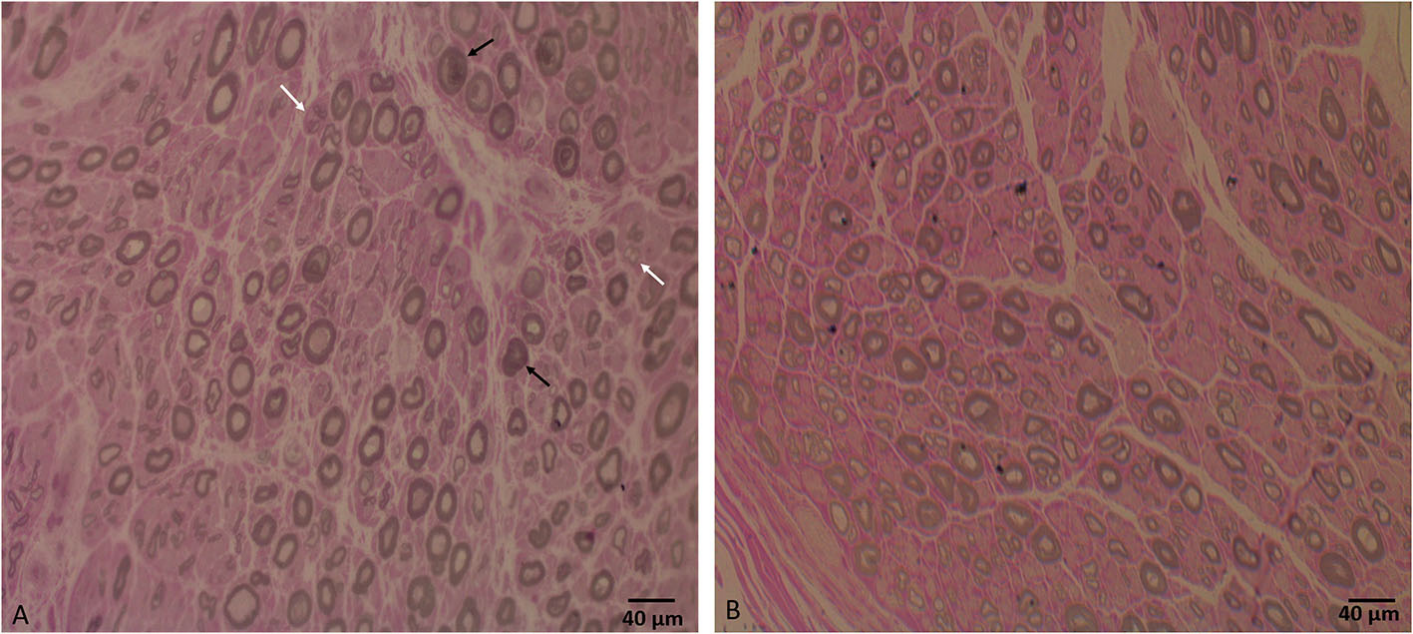
**(A)** Sural nerve biopsy semithin section shows a moderate decrease in large, myelinated fiber density for the patient's age, axonal degeneration (black arrows), and regeneration profiles (white arrows). **(B)** Semithin section of a normal sural nerve biopsy processed at our laboratory. Bar = 40 μm.

**Figure 2:**
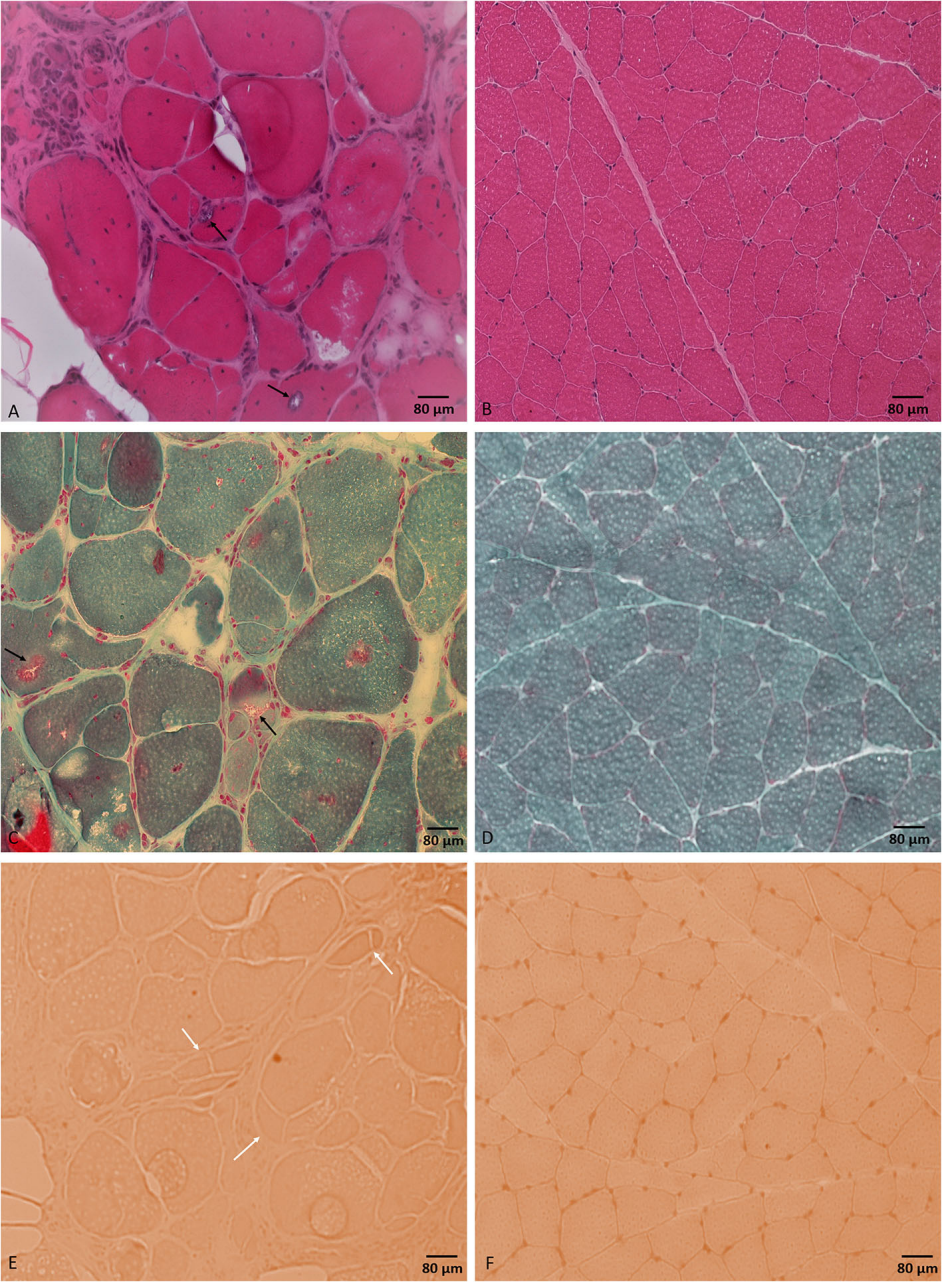
Tibialis anterior muscle biopsy. H&E stained section **(A)** shows different myopathic features including marked variability in muscle fiber diameters and muscle fiber necrosis, regeneration, and splitting. Muscle fibers are harboring internalized nuclei and rimmed vacuoles (black arrows). Modified Gomori trichrome stained section **(C)** shows rimmed vacuoles (black arrows). Congo red stained section **(E)** shows prominent fiber splitting (white arrows). No inflammatory changes were noticed throughout the sample. **(B, D, F)** represent normal control muscle biopsy-stained sections by H&E, modified Gomori trichrome, and Congo red, respectively, processed at our laboratory. Bar = 80 μm.

Laboratory work up was notable for an elevated creatine kinase level ranging between 527 and 1019 units/L (with a reference range of 35–250 units/L). The complete blood count and comprehensive metabolic panel were normal. Rheumatologic workup showed an elevated anti-Smith antibody (28 units, with normal range < 19 units) with a negative antinuclear antibody. An inflammatory myopathy antibody panel showed a positive OJ IgG antibody and anti-SRP antibody, and the latter was negative when repeated. Nerve conduction studies of upper and lower limbs demonstrated normal sensory responses with reduced lower limb compound muscle action potential amplitudes and normal conduction velocities (Table 1). Needle electromyography revealed positive sharp waves and fibrillation potentials in the distal arm and leg muscles (right first dorsal interosseous, tibialis anterior, and gastrocnemius muscles) and reduced recruitment of long-duration motor unit potentials in both the distal and proximal muscles (the right first dorsal interosseous, deltoid, tibialis anterior, gastrocnemius, and vastus lateralis muscles). There were no short-duration motor unit potentials, myotonia, myokymia, or neuromyotonia in any tested muscle. Sural nerve and tibialis anterior muscle biopsies were obtained by the referring neurologist before the patient was seen in our clinic. The former showed a moderately decreased density of myelinated fibers, increased rates of axonal degeneration, and rare regeneration profiles, which is consistent with a non-specific chronic axonal neuropathy (Figure 1). A biopsy of the tibialis anterior muscle (Figure 2) demonstrated marked variability in myofiber size diameters, muscle fiber necrosis, regenerating fibers, internalized nuclei, and fiber splitting in addition to several muscle fibers harboring rimmed vacuoles without inflammation. Rimmed vacuoles were observed in up to two fibers per fascicle. The endomysial fibrous connective tissue also increased. Mild chronic denervation changes were noticed including few angular atrophic fibers of both histochemical fiber types and fiber type grouping.

**Table 1:** Nerve conduction studies (NCS).

**Motor NCS**	**Latency (ms)**	**Amplitude (mV)**	**NCV (m/s)**
**Right peroneal (EDB)**			
Ankle	**NR**		
Below fibular head	**NR**		
Popliteal fossa	**NR**		
**Right peroneal (TA)**			
Fibular head	3.5 ( ≤ 6.8)	**2.7** (≥ 4)	
Popliteal fossa		**2.2**	40 (≥40)
**Right tibial**			
Ankle	4.1 ( ≤ 5.1)	**0.5** (≥6)	
Knee		**0.4**	42 (≥40)
**Right median**			
Wrist	**3.8** ( ≤ 3.5)	9.0 (≥7)	
Elbow		8.6	52 (≥50)
**Right lnar**			
Wrist	3.0 ( ≤ 3.0)	**6.9** (≥7)	
Below elbow		5.9	53 (≥50)
Above elbow		5.1	5025.8pt
**Sensory antidromic NCS**	**Latency (ms)**	**Amplitude (**μ**V)**	**NCV (m/s)**
**Right superficial peroneal**			
Dorsal foot	3.3 ( ≤ 3.7)	11.0 (≥ 4)	50 (≥40)
**Right sural**			
Calf	3.5 ( ≤ 4.3)	13.3 (≥6)	50 (≥40)
**Right median (2nd digit)**			
Wrist	2.3 ( ≤ 3.5)	24.9 (≥20)	57 (≥50)
**Right ulnar**			
Wrist	3.2 ( ≤ 3.4)	24.7 (≥18)	50 (≥50)
**Right radial (Base 1st digit)**			
Wrist	2.5 ( ≤ 3.1)	27.5 (≥20)	57 (≥50)

Values shown in bold font are abnormal. EDB, extensor digitorum brevis; m/s, meter per second; μV, microvolt; ms, milli seconds; mV, millivolt; NR, no response; NCV, nerve conduction velocity; TA, tibialis anterior. The number in the parentheses indicates the normal value.

A comprehensive genetic testing panel for inherited neuropathies performed in both our patient and his brother showed the same homozygous missense variant in the coding exon 2 of *HINT1* gene p.I63N (c.188T > A) in both siblings. While the *in silico* prediction was inconclusive, the specific encoded amino acid (isoleucine) was reported to be well-conserved in vertebrate species, and this variant may not be found in the healthy population. In addition, we found two heterozygous variants of unknown significance, one in the *NGF* gene, c.482G > C (population frequency in Africans/African Americans according to the gnomAD database is 0.008%) and another variant in the *SETX* gene,c.5473A > G (population frequency in Africans/African Americans is 0.164%). These are associated with autosomal recessive hereditary sensory and autonomic neuropathy type V and autosomal dominant juvenile amyotrophic lateral sclerosis, respectively, none of which fits his clinical picture. A comprehensive neuromuscular genetic panel obtained in the proband also reported another heterozygous variant of unknown significance in *AMPD1*, c.1162C > T, and p.Arg421Trp (population frequency in Africans/African Americans is % 0.713) missense variant in the patient, but not tested in his sibling (see Supplementary material). Specific genetic testing for spinal muscular atrophy showed normal *SMN1/SMN2* gene analysis. Our patient received supportive care including orders for physical therapy, ankle foot orthosis, and a rolling walker.

## Discussion

Since the initial report of eight mutations in the *HINT1* gene that were associated with autosomal recessive axonal motor-predominant CMT with neuromyotonia in 2012 (8), twenty-four different causal *HINT1* mutations have been described worldwide. In this study, we report a novel, likely pathogenic, a homozygous variant in *HINT1* gene p.I63N (c.188T>A) not previously included in public databases. The clinical manifestations of this patient are similar to previously reported cases, including the insidious onset of progressive symmetric distal muscle weakness and atrophy as well as foot and hand deformities without sensory involvement on clinical examination. Electrodiagnostic testing showed a motor axonal polyneuropathy but a sural nerve biopsy demonstrated sensory involvement as well. Furthermore, his brother who has similar but less severe clinical features (no more details provided due to lack of consent from the brother) also had the same homozygous *HINT1* variant. Based on the clinical presentation, molecular genetic analyses, and family history, a *HINT1*-related dHMN/motor-predominant polyneuropathy due to a novel p.I63N (c.188T>A) mutation was diagnosed in this patient.

Neuromyotonia represents high-frequency (150–220Hz) repetitive firing of a single motor unit. Clinically, it manifests with muscle stiffness, cramping/pain, and delayed muscle relaxation after contraction. It was reported in 70–80% of patients with *HINT1* and was considered a diagnostic hallmark of the disease (2). Our patient denied significant muscle cramps, stiffness, and no trouble with muscle relaxation. He had no evidence of neuromyotonia in electrodiagnostic studies. *HINT1* mutation-related motor-predominant axonal neuropathies both with and without neuromyotonia have been reported with widespread geographical distribution, including Central and South-East Europe (2, 8, 9); Russia and Scandinavia (5, 10, 11); China (6, 12); and South America (13) and North America in a 30-year-old man of Slovenian heritage (14). Here, we report another case of dHMN/motor-predominant axonal neuropathy due to a novel *HINT1* mutation in an African American family. Our findings broaden the genetic epidemiology of *HINT1*-neuropathy and have implications for molecular diagnostics of inherited peripheral neuropathies in African Americans with no apparent European ancestry.

A tibialis anterior muscle biopsy showed non-specific chronic myopathic changes and several muscle fibers harboring rimmed vacuoles with no inflammation. Rimmed vacuoles are small areas of focal destruction of muscle fibers, found in inclusion body myositis, myofibrillar myopathies, and certain distal myopathies (15). The myopathic changes seen in muscle biopsy are unlikely to be explained by the detected variant of unknown significance in the *AMPD1* gene as cases of autosomal recessive myoadenylate deaminase deficiency typically present with rhabdomyolysis with normal muscle biopsy. We only found one case report in the literature of an individual with myopathy and a compound heterozygous *AMPD1* variants including the same mutation, c.1162C > T (p.Arg421Trp), in addition to another mutation, c.1274G > A (p.Arg425His) (16). The positive OJ IgG, the one-time positive anti-SRP, and the anti-Smith antibodies are of unclear clinical significance in the setting of negative ANA and the muscle biopsy lacking evidence of inflammation. Our patient's creatinine kinase (CK) level was mild to moderately elevated, which was consistent with the previous reports in patients with *HINT1* (2, 8, 12, 13). Based on the significant myopathic findings in our case's muscle biopsy and the elevated CK levels, the limb weakness in our patient likely represented a combined neuropathic and myopathic involvement. Although the significance of these findings remains uncertain, it does raise the possibility that *HINT1* gene mutations may be associated with myopathy in addition to neuropathy.

The identified *HINT1* mutation is very close in exon location to another previously reported mutation, c. 182C>T, p. Gln62^*^ (compound heterozygous with c. 278G>A, p. Gly93Asp) which was reported to cause a phenotype of distal hereditary motor neuropathy in two patients from the same family (8). The neuropathic mechanisms triggered by *HINT1* mutations are still unclear. HINT1 interacts with the DNA helicases Pontin and Reptin, thereby modulating the β-catenin transcriptional activity, which is essential for the wnt/ β-catenin pathway that regulates Schwann cells migration and proliferation (17). Pontin and Reptin also form a complex with IGHMBP2, which is implicated in an autosomal recessive type of distal motor neuropathy with respiratory distress (SMARD1) (18). In addition, HINT1 binds to lysyl-tRNA synthetase and hydrolyzes the lysyl-AMP that is generated by this enzyme. Similarly, HINT1 was suggested to regulate the activity of other aminoacyl-tRNA synthetases, a protein family of four members (GlyRS, TyrRS, AlaRS, and LysRS), which are directly implicated in hereditary peripheral neuropathies (19).

HINT1 protein is widely expressed in the central nervous system and in other tissues (20, 21). It is critical to maintaining the normal function of motor neurons (22). It establishes a series of regulatory interactions with signaling proteins enriched in motor neurons, such as the type 1 sigma receptor or intracellular domain of transmembrane teneurin 1, both of which are also implicated in motor dysfunction. HINT1 also can remove the small ubiquitin-like modifier from a series of substrates that could be affected by *HINT1* mutations (23). HINT1 is also implicated in the regulation of mood and behavior, suggesting an additional role in the central nervous system (2). HINT1 levels are increased in the dorsolateral prefrontal cortex of patients with major depression disorder (24), and it was reported that a *HINT1* neuropathy patient developed psychiatric symptoms (25). In our patient, no abnormal behavior or psychiatric abnormality was noticed.

Despite the increasing number of patients with *HINT1* neuropathy being diagnosed worldwide, it remains challenging to assess the pathogenicity of novel *HINT1* variants. We were not able to get the whole genome sequencing of the proband. However, in the absence of unbiased whole genome sequencing of the proband, the discovered likely pathogenic mutation coupled with the patient's clinical phenotype, based on the used genotyping platforms, strongly suggests this homozygous *HINT1* mutation to be causative/contributing to the neuropathy. We concluded that this variant was most likely pathogenic and contributed to neuropathy. With the majority of the *HINT1*-related disorder case reports, including ours, denoting the elevation of CK, the presence of rimmed vacuoles in a muscle biopsy taken from our case raises the possibility that *HINT1* mutations can cause myopathy in addition to neuropathy. The earlier case studies did not include muscle biopsy findings, and further investigations are required to establish a more solid relationship.

## Data availability statement

The original contributions presented in the study are included in the article/Supplementary material, further inquiries can be directed to the corresponding author.

## Ethics statement

Ethical review and approval was not required for the study on human participants in accordance with the local legislation and institutional requirements. The patients/participants provided their written informed consent to participate in this study.

## Author contributions

NJ: writing and editing the original draft and table. RV: discussing the case, reviewing, and editing the draft. MK: preparing the figures, reviewing, and editing the draft. All authors contributed to the article and approved the submitted version.
