# Supplementary material for: Case report: A novel homozygous histidine triad nucleotide-binding protein 1 mutation featuring distal hereditary motor-predominant neuropathy with rimmed vacuoles
Source: Front Neurol. 2023 Feb 6;14:1007051. doi: 10.3389/fneur.2023.1007051 (PMC9943687; doi:10.3389/fneur.2023.1007051)
Supplement: Supplementary file 1 [file Data_Sheet_1.PDF]

Supplementary file

1. Comprehensive neuromuscular disease panel by Perkin Elmer genomics testing the following genes:

ACTA1, ADSSL1, AMPD1, ANO5, ATP2A1, B3GALNT2, BAG3, BIN1, BVES, CAPN3, CAV3, CFL2, CHAT, CHRNA1, CHRNB1, CHRND, CHRNE, CLCN1, CNBP, COL12A1, COL6A1, COL6A2, COL6A3, COLQ, CRYAB, DAG1, DES, DMD, DMPK, DNAJB6, DNM2, DOK7, DPM3, DYSF, EMD, FHL1, FKRP, FKTN, FLNC, GAA, GMPPB, GNE, GOSR2, HNRNPA1, HNRNPDL, HSPG2, IGHMBP2, ISPD, ITGA7, LAMA2, LARGE, LDB3, LIMS2, LMNA, LYST, MOD3, LRP4, MATR3, MRE11, MTM1, MTMR14, MUSK, MYH2, MYH7, MYOT, NEB, PABPN1, PLEC1, PNPLA2, POGLUT1, POMGNT1, POMK, POMT1, POMT2, PTRF, PYGM, RAPSN, RNPA2, RYR1, SCN4A, SELENON, SGCA, SGCB, SGCD, SGCG, SIL1, SMCHD1, SYNE1, SYNE2, TAZ, TCAP, TIA1, TNNT1, TNPO3, TOR1AIP1, TPM2, TPM3, TRAPPC11, TRIM32, TTN,VCP

2. The hereditary neuropathy panel by Ambry testing the following genes:

AARS, AIFM1, APOA1, ATL1, ATL3, ATP7A, BICD2, BSCL2, CHCHD10, DCTN1, DNAJB2, DNM2, DNMT1, DST, DYNC1H1, EGR2, FAM134B, FBXO38, FGD4, FIG4, FUS, GAN, GARS, GDAP1, GJB1, GNB4, GSN, HARS, HINT1, HSPB1, HSPB8, IGHMBP2, IKBKAP, INF2, KIF1A, LITAF, LMNA, LRSAM1, MARS, MFN2, MORC2, MPZ, MTMR2, NDRG1, NEFH, NEFL, NGF, NTRK1, OPTN, PDK3 (c.473G>A[p.R158H]), PLEKHG5, PMP22, PRDM12, PRPS1, PRX, RAB7A, REEP1, SBF2, SCN10A, SCN11A, SCN9A, SETX, SH3TC2 , SIGMAR1, SLC25A46, SLC52A2, SLC52A3, SLC5A7, SPG11, SPTLC1, SPTLC2, TARDBP, TFG, TRPV4, TTR, UBA1, VAPB, VCP, VRK1, WNK1, and YARS.

The mutation results of the above neuromuscular disease and hereditary neuropathy panels

Gene	DNA change	Protein change	Zygosity	Classification	Frequency in African/ African Americans*
HINT1	c.188T>A	p.I63N	homozygous	Uncertain significance	Novel
NGF	c.482G>C	p.Gly161Ala	Heterozygous	Uncertain significance	0.00008010
SETX	c.5473A>G	p.Thr1825Ala	Heterozygous	Uncertain significance	0.001643
AMPD1	c.1261C>T	p.Arg421Trp	Heterozygous	Uncertain significance	0.007131
PNPLA2	c.785T>G	p.Leu262Arg	Heterozygous	Uncertain significance	0.00007349
RYR1	c.2861T>C	p.Leu954Pro	Heterozygous	Uncertain significance	0.0†
SYNE1	c.1412A>G	p.His471Arg	Heterozygous	Uncertain significance	0.002
TTN	c.397C>G	p.Pro133Ala	Heterozygous	Uncertain significance	Novel
TTN	c.85052G>A	p.Arg28351Gln	Heterozygous	Uncertain significance	0.000044
GAA	c.2065G>A	p.Glu689Lys	Heterozygous	Pseudodeficiency allele	0.01975

* Based on the Genome Aggregation Database, gnomAD v2.1.1

† Frequency in European (non-Finnish) population according to the gnomAD database is 0.00001949 and total population is 0.000008646.
